# T3SEpp: an Integrated Prediction Pipeline for Bacterial Type III Secreted Effectors

**DOI:** 10.1128/mSystems.00288-20

**Published:** 2020-08-04

**Authors:** Xinjie Hui, Zewei Chen, Mingxiong Lin, Junya Zhang, Yueming Hu, Yingying Zeng, Xi Cheng, Le Ou-Yang, Ming-an Sun, Aaron P. White, Yejun Wang

**Affiliations:** a Department of Cell Biology and Genetics, School of Basic Medicine, Shenzhen University Health Science, Shenzhen, China; b Guangdong Key Laboratory of Intelligent Information Processing, Shenzhen Key Laboratory of Media Security, College of Information Engineering, Shenzhen University, Shenzhen, China; c College of Veterinary Medicine, Yangzhou University, Yangzhou, China; d VIDO-InterVac, University of Saskatchewan, Saskatoon, Saskatchewan, Canada; Pacific Northwest National Laboratory

**Keywords:** effector, machine learning, prediction, T3SEpp, T3SS, type III secretion system

## Abstract

Many Gram-negative bacteria infect hosts and cause diseases by translocating a variety of type III secreted effectors (T3SEs) into the host cell cytoplasm. However, despite a dramatic increase in the number of available whole-genome sequences, it remains challenging for accurate prediction of T3SEs. Traditional prediction models have focused on atypical sequence features buried in the N-terminal peptides of T3SEs, but unfortunately, these models have had high false-positive rates. In this research, we integrated promoter information along with characteristic protein features for signal regions, chaperone-binding domains, and effector domains for T3SE prediction. Machine learning algorithms, including deep learning, were adopted to predict the atypical features mainly buried in signal sequences of T3SEs, followed by development of a voting-based ensemble model integrating the individual prediction results. We assembled this into a unified T3SE prediction pipeline, T3SEpp, which integrated the results of individual modules, resulting in high accuracy (i.e., ∼0.94) and >1-fold reduction in the false-positive rate compared to that of state-of-the-art software tools. The T3SEpp pipeline and sequence features observed here will facilitate the accurate identification of new T3SEs, with numerous benefits for future studies on host-pathogen interactions.

**IMPORTANCE** Type III secreted effector (T3SE) prediction remains a big computational challenge. In practical applications, current software tools often suffer problems of high false-positive rates. One of the causal factors could be the relatively unitary type of biological features used for the design and training of the models. In this research, we made a comprehensive survey on the sequence-based features of T3SEs, including signal sequences, chaperone-binding domains, effector domains, and transcription factor binding promoter sites, and assembled a unified prediction pipeline integrating multi-aspect biological features within homology-based and multiple machine learning models. To our knowledge, we have compiled the most comprehensive biological sequence feature analysis for T3SEs in this research. The T3SEpp pipeline integrating the variety of features and assembling different models showed high accuracy, which should facilitate more accurate identification of T3SEs in new and existing bacterial whole-genome sequences.

## Abstract (precis)

Type III secreted effector (T3SE) prediction remains a big computational challenge. In practical applications, current software tools often suffer problems of high false-positive rates. One of the causal factors could be the relatively unitary type of biological features used for the design and training of the models. In this research, we made a comprehensive survey on the sequence-based features of T3SEs, including signal sequences, chaperone-binding domains, effector domains, and transcription factor binding promoter sites, and assembled a unified prediction pipeline integrating multi-aspect biological features within homology-based and multiple machine learning models. To our knowledge, we have compiled the most comprehensive biological sequence feature analysis for T3SEs in this research. The T3SEpp pipeline integrating the variety of features and assembling different models showed high accuracy, which should facilitate more accurate identification of T3SEs in new and existing bacterial whole-genome sequences.

## INTRODUCTION

The type III secretion system (T3SS) is a complex needle-like nanomachine used by many Gram-negative bacteria to interact with host cells (1, 2). A variety of bacterial virulence proteins can be recognized and delivered into the host cell cytoplasm via T3SS conduits (3). These virulence proteins are called type III secreted effectors (T3SEs), which are often enzymes that cause a sequence of host cell changes and disease (2, 4). The number, function, and sequences of T3SEs vary significantly among different T3SSs or bacterial species. Only a small subset of T3SEs has been disclosed for most bacterial strains with a functional T3SS (5). Thus, it remains important to identify new effectors, which together play essential roles in the establishment of infection for many pathogens.

It is a difficult challenge to identify new T3SEs, since they lack sequence conservation or typical common features (6, 7). The first T3SEs were identified experimentally due to their location adjacent to T3SS apparatus genes (8, 9). Other features were also used for T3SE identification, for example, common motifs in signal peptides (10, 11) and proximity to genes encoding chaperones (12). In 2009, two bioinformatic tools were published to predict T3SEs (13, 14). Since then, machine learning algorithms have been introduced to the field (15–19), with new features or feature representation schemes added to further improve their performance (20–23). Most recently, a deep convolutional neural network (CNN) model was also proposed to predict T3SEs (24). Despite the promise of machine learning strategies, their practical significance remains to be evaluated. Only a few T3SEs have been identified with the assistance of these tools (14, 25).

Most T3SEs have been identified based on homology searching of known effectors, despite the “common view” that there is low conservation between T3SEs (8, 10, 11). An N-terminal reassortment hypothesis was proposed to explain the evolution of T3SEs and their signal sequences (26), and the complete conservation of signal sequences was further used to screen new T3SEs (27). Conserved motifs present in effector domains of T3SEs, e.g., EPIYA and LPX, have been discovered and used successfully for prediction of new T3SEs (28, 29). Conservation was also found in the promoter regions for effector genes, and several T3SEs were successfully predicted using this feature (30, 31). In a previous study, we predicted more than 8,000 new T3SEs from diverse bacteria based on full-length homology searching against a comprehensive list of validated effectors, further indicating the power of homology searching in prediction of new T3SEs (5).

Homology-based or machine learning algorithms for detection of T3SEs each have their own drawbacks. It would be meaningful to design an integrated prediction pipeline to consider the multi-aspect biological features and algorithms comprehensively and take the advantages but avoid the limitations of each model. Recently, computational biologists have realized the importance of integrating both strategies. For example, pEffect (32) and BEAN2 (21) can implement both homology searching and machine learning algorithms to increase the prediction sensitivity. However, a simple combination did not relieve the false-positive problem for the results of machine learning methods. Most recently, Wang and colleagues proposed a two-layer ensemble T3SE predictor, which considered multiple features, including the two arms stated above and integrated them in a general hierarchical model (23). The method was reported to reach significantly improved performance compared to that of other tools (23).

In this research, we also proposed a new hybrid strategy but integrated more comprehensive biological features for analysis and T3SE prediction. First, a full-length protein-, domain-, and gene promoter-based pipeline was developed based on the homology among known effectors and signal sequences. This pipeline was evaluated to screen effector candidates of either global or local homology within known families. Furthermore, we integrated machine learning modules into this prediction pipeline, which was used to predict novel candidates without homology to known effectors. For the machine-learning modules, previously developed BPBAac (16) and T3_MM (18) were updated with the latest version of manually annotated effector data and phylogenetic information on T3SSs and bacterial species (5). Recently, deep learning techniques have been successfully introduced to models predicting the subcellular localization of proteins (33, 34). In fact, two CNN-based T3SE prediction models were proposed recently (24). Here, we have developed an integrated prediction pipeline employing two different deep learning models based on the signal sequences of T3SEs and taking into account various biological properties of candidate proteins, for example, the presence or absence of a transmembrane domain or classical signal peptide. Each module was weighted, and a general score was generated from a linear model, to reflect the likelihood of a protein being a T3SS effector.

## RESULTS

### Sequence homology among verified effectors and the integrated prediction framework.

**FIG 1:**
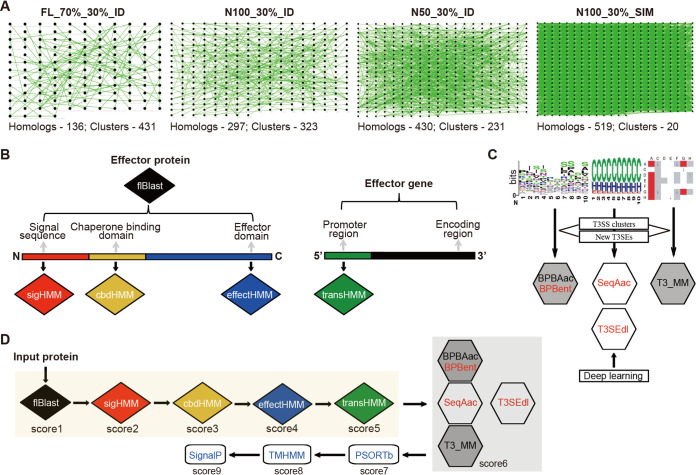
Sequence homology among T3Enc effectors and the integrated prediction framework. (A) Sequence homology network of T3Enc-verified effectors. The nodes represented effectors with homology with at least one other effector. The pairs with homology (identified by the criteria defined at the top) were connected by green lines. (B) Homology-based modules developed for T3SEpp, based on the full-length effector proteins (flBlast) or signal sequence (sigHMM), chaperone binding (cbdHMM), and effector (effectHMM) domains or based on the promoter region of the effector genes (transHMM). (C) The machine learning models that were preexisting but have been improved with updated training effectors (black font with gray background), newly developed with similar feature representation schemes (red font with gray background), or developed with new algorithms (red font with white background) are shown. T3SEdl has two deep learning models, i.e., T3SEdnn and T3SErnn. (D) Procedural flowchart for T3SEpp prediction. The weighted sum of the prediction score for each individual module is incorporated into the probability that a protein is a T3SE.

The T3Enc data set contains 519 manually annotated effectors, representing the newest and most comprehensive list of experimentally validated T3SEs (see Materials and Methods) (5). Pairwise sequence alignment was performed for the full-length (FL) effector proteins or their N-terminal peptides of 100 or 50 amino acids (N100 or N50, respectively). For FL proteins, with a relatively strict homology measure (i.e., larger than 30% identity [ID] for longer than 70% aligned fragments), 431 nonhomologous clusters were identified after homology filtering (Fig. 1A) (FL_70%_30%_ID). When N100 sequences were analyzed, however, 297 homologs were identified with larger than 30% identity, and 323 nonredundant clusters were retained (Fig. 1A) (N100_30%_ID). This reduced number of clusters showed that there was more homology in the N-terminal 100 amino acids than in the full-length effector proteins (EBT *P *= 1.8e−7). The N50 sequences further reflected the more typical N-terminal homology among effectors: 430 peptides were found with homology among each other, while only 231 clusters remained after homology filtering (Fig. 1A) (N50_30%_ID, 323/519 versus 231/519, EBT *P *= 6.0e−5). For machine learning applications in sequence analysis and effector recognition, strict homology filtering is a prerequisite. The sequence homology is often measured with similarity (SIM) instead of identity, and the cutoff is set as 30% or lower. Therefore, a loosened homology measure was also adopted to examine the sequence similarity among the verified effectors, with homology defined as >30% similarity. Surprisingly, all 519 N100 peptides showed homology with at least one other peptide. After filtering, only 20 clusters remained that did not show homology with each other (Fig. 1A) (N100_30%_SIM). Taken together, the results demonstrated that the verified T3SEs showed unexpected striking homology between each other, with higher homology near the N termini.

An integrated prediction pipeline (T3SEpp) was designed, taking full advantage of the fragmental similarity among T3SEs, together with machine learning techniques (Fig. 1B and C). We developed several modules to identify homologues of full length (flBlast), N-terminal signal regions (sigHMM), chaperone-binding domains (cbdHMM), effector domains (effectHMM), and putative T3 transcription factor binding promoter sites (transHMM) of known T3SEs. Previous machine learning models such as BPBAac, SeqAac, and T3_MM were improved by integrating information from T3SS clusters, newly validated effectors, and new algorithms (16, 18). A new prediction module, T3SEdl, was developed to use deep learning algorithms and new features. An example was given to illustrate the T3SEpp prediction framework (Fig. 1D). The modules described above (flBlast, sigHMM, cbdHMM, effectHMM, and transHMM) were applied and fragmental scores were calculated, followed by analysis of machine learning modules and other established protein subcellular localization tools, e.g., PSORTb, TMHMM, and SignalP (34–36). The final probability was calculated by integrating the prediction results of all steps, which represented the likelihood of an input protein to be an effector.

### Signal sequence families and homology screening of T3SEs.

Thousands of T3SE candidates were detected based on the homology with the full-length protein sequences of known effectors with flBlast (see Materials and Methods) (5). However, the proteins that have general homology with known effectors could have lost the N-terminal signal sequence that is necessary to be recognized by T3SSs. On the other hand, new effector proteins could have type III secretion (T3S) signal sequences but lack general sequence similarity with known effectors (27). Therefore, we reasoned that homology searching based on signal sequences would facilitate the identification of T3SEs.

**FIG 2:**
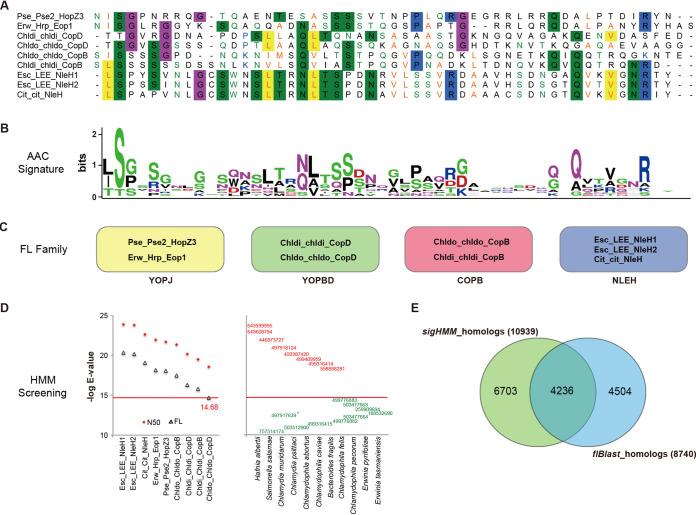
Effector screening based on the fragmental homology with signal sequences of verified T3SS effectors. (A) Multiple-sequence alignment of a homologous cluster (i.e., SigFAM_3) of T3SS effector signal sequences. The N-terminal 50 amino acids, after the starting M was removed, were retrieved from each effector and aligned. Gaps are represented with “-.” The positions with >40% of sequences showing identical amino acid composition were highlighted with uniformly colored backgrounds, while the positions without a highlighted background are shown in uniformly colored font, for which more than 40% of sequences showed amino acid compositions with conserved physicochemical properties. (B) Sequence logo of the position-specific amino acid composition (AAC) corresponding to the alignment shown in panel A. The height of the amino acid in each position indicated the AAC preference. (C) Family clustering of the corresponding full-length (FL) effectors of SigFAM_3 members. (D) Similarities of SigFAM_3 members (N50) and their FL effectors (FL) aligned against the HMM profile built with SigFAM_3 member sequences (left), and the new proteins predicted with sequence similarity with the HMM profile of SigFAM_3 (right). The lowest similarity score of the known FL SigFAM_3 member is indicated, which represented the strict cutoff. Predicted proteins with higher similarity scores than the strict cutoff are shown in red, and those with lower similarity are shown in green, with GI accession numbers shown. *, protein was from a different strain of the corresponding species. (E) Venn diagram showing proteins predicted with N-terminal sequence HMM profiles only (6,703), full-length homology only (4,504), or with both sequence features (4,236).

An alignment-based clustering strategy grouped the N-terminal 50 amino acids of the 519 verified effectors into 379 signal sequence families, with 84 multicomponent and 295 singleton families (see Data Set S1, sheet 1, in the supplemental material). The sequences within each multicomponent family showed striking similarity, and multiple positions appeared conserved, as shown for one example, SigFAM_3 (Fig. 2A). The amino acid composition (AAC) showed apparent preference in multiple positions, e.g., serines in positions 3, 6, 22, and 23, glutamine in position 43, and asparagine in position 49 of SigFAM_3 (Fig. 2B). Despite the homology among the N50 signal sequences within each signal family, the effectors were classified into different groups based on full-length protein homology clustering (Fig. 2C). Effectors from other signal families displayed the same trends (data not shown).

10.1128/mSystems.00288-20.4 DATA SET S1 T3SE clusters based on the conservation of fragmental features. Download Data Set S1, XLSX file, 4.0 MB. Copyright © 2020 Hui et al. 2020 Hui et al. This content is distributed under the terms of the Creative Commons Attribution 4.0 International license.

A profile hidden Markov model (HMM) was built for each signal sequence family. The similarity cutoff was determined by aligning the signal sequence-containing effectors (full length or N50) against the corresponding HMM profile (Fig. 2D, left; for the example SigFAM_3). Effector prediction can be conducted with the similarity cutoffs determined. For example, the SigFAM_3 HMM was able to detect 8 new proteins with high homology and 10 with intermediate homology in 11 species with putative T3SSs (Fig. 2D, right). It should be noted that signal sequence homology was only detected in the N-terminal region and not elsewhere in the protein.

Screening with the HMMs for all signal families detected 10,939 T3S signal homologs from the proteome of strains with putative T3SSs (Data Set S1, sheet 2): 4,236 were detected with flBlast for full-length homologs and 6,703 (61.2%) were only found with T3S signal sequences. Meanwhile, 4,504 (51.5%) full-length effector homologs were not detected with putative T3S signal sequences (Fig. 2E).

### Sequence signatures of chaperone-binding domains.

**FIG 3:**
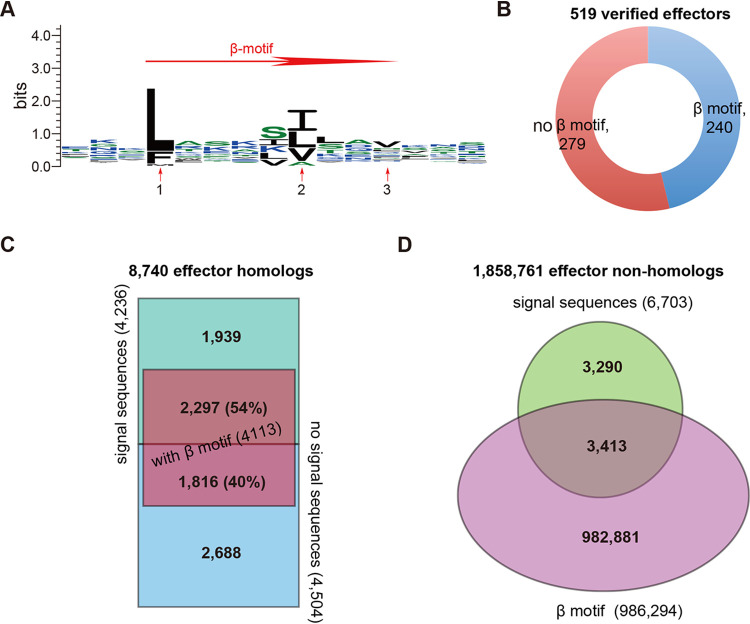
β-Motifs of chaperone-binding domains and CBD-based effector screening. (A) WebLogo-based signature of the position-specific amino acid compositions of β motifs. The key positions with conserved physicochemical properties are indicated with arrows. (B) Distribution of β-motifs in 519 verified effectors. The distributions of β-motifs in full-length effector homologs with or without putative T3SS-recognized signal sequences (C) and in nonhomologs of full-length known effectors with or without putative T3SS-recognized signal sequences (D).

Successful translocation of T3SEs is often dependent on the binding of chaperone proteins. The mechanisms by which effectors and chaperones interact have not been fully clarified yet. However, structural signatures have been identified in the chaperone-binding domains (CBDs) of effectors, such as a conserved β-motif (37). Sequence alignment of the known β-motifs disclosed striking amino acid composition preference in some positions of the motif, e.g., L and F in position 1 and I, L, or V in position 2 (Fig. 3A).

An HMM profile was modeled for the β-motif of T3SE CBDs, followed by screening for homologs of the verified effectors. In total, ∼46% (240/519) of the effectors contained the motif located between possible translocation signal sequences and effector domains (Fig. 3B; Data Set S1, sheet 3). The motifs screened by the HMM profile showed conserved amino acid composition in key positions and a similar β helix secondary structure as predicted with PROMALS3D (see Fig. S1). Previous tertiary structure analysis also demonstrated the similar three-dimensional (3D) conformation of the motifs (37). Similar percentages of the full-length effector homologs (47%= [4,113/8,740]) contained the β-motif within possible CBDs, especially those also with putative T3SS-recognized signal sequences (54% [2,297/4,236]) (Fig. 3C; Data Set S1, sheet 3). β-Motifs were also detected in 3,413 proteins that were not general homologs of known effectors but contained putative T3S signals (Fig. 3D; Data Set S1, sheet 3).

10.1128/mSystems.00288-20.1 FIG S1 Conservation of amino acids (AA) and secondary structure (SS) of the representative β-motifs in verified effectors. Conserved positions are highlighted. *h*, hydrophobicity; e, strand. Download FIG S1, PDF file, 0.1 MB. Copyright © 2020 Hui et al. 2020 Hui et al. This content is distributed under the terms of the Creative Commons Attribution 4.0 International license.

### Function domain families present in verified T3SS effectors.

**FIG 4:**
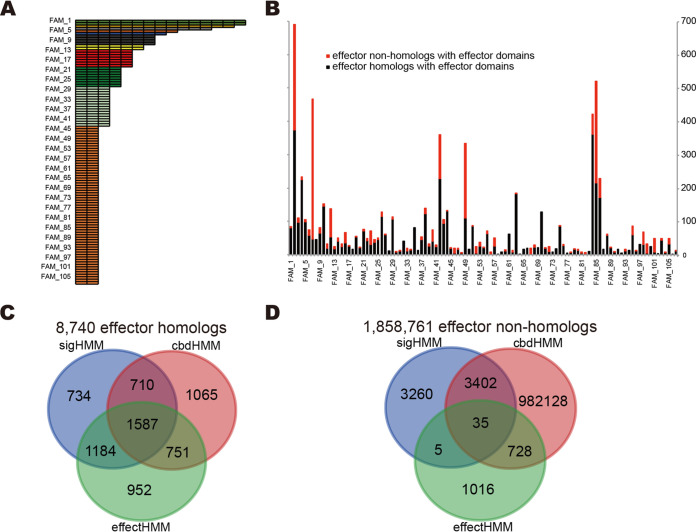
Effector domain families of know effectors. (A) Schematic of effector domain families showing the number of members in each family; the individual members of each family are listed in Data Set S1, sheet 4, in the supplemental material. (B) Distributions of proteins with putative effector domains in full-length homologs (black) and nonhomologs (red). The family accessions are listed in increasing order along the horizontal axis, and only the odd numbers are shown due to the space limitation. Interplay of proteins with T3SS signal sequences, effector domains, and β-motifs in full-length effector homologs (C) and in nonhomologs of full-length known effectors (D).

After removal of the signal sequences (N50) for all effector proteins in the T3Enc set, 508 verified effectors remained with length >30 amino acids, among which 356 were classified into 107 multimember families and 152 were singletons (Fig. 4A; Data Set S1, sheet 4). The most widely distributed families included YopJ and YopM (EffectFAM_1 and_2; 15 members), YopH (EffectFAM_3; 14 members), and YopB (EffectFAM_4; 12 members) (Fig. 4A; Data Set S1, sheet 4). Different effector families also showed varied T3S signals, consistent with Fig. 2C. For example, 13 of 15 YopJ effector family members classified into 13 different signal families (Data Set S1, sheet 5). Only a small proportion of the validated effectors showed conservation for both signals and effector domains; the NleB family in Escherichia coli, *Citrobacter*, and *Salmonella* (i.e., Effect_FAM8) had 5 of 7 effectors also in the SigFAM_7 T3S signal pattern group (Data Set S1, sheet 5). An HMM profile was built for each multimember family and used to screen different protein data sets. Half of the full-length effector homologs (4,474/8,740 [51%]) contained effector domains that belonged to the multimember families (Fig. 4B; Data Set S1, sheet 6). Surprisingly, the domains were also detected in 1,784 proteins that were not identified by flBlast as having homology to full-length effectors (Fig. 4B; Data Set S1, sheet 6). From the 8,740 full-length effector homologs, 2,771 (32%) contained both T3S signal sequences and effector domains (Fig. 4C; Data Set S1, sheet 6). One thousand five hundred eighty-seven (18%) of the full-length homologs contained all three features: signal sequences, the chaperone binding β-motif, and effector domains (Fig. 4C; Data Set S1, sheet 6). From the 1,858,761 proteins in bacterial strains with T3SSs that were not homologues of known effectors, only 40 proteins contained both T3S signal sequences and putative T3SS effector domains, and 35 of these also contained β-motifs (Fig. 4D; Data Set S1, sheet 6).

### Consensus motifs within regulon promoters of pivotal T3SS transcription regulators.

**FIG 5:**
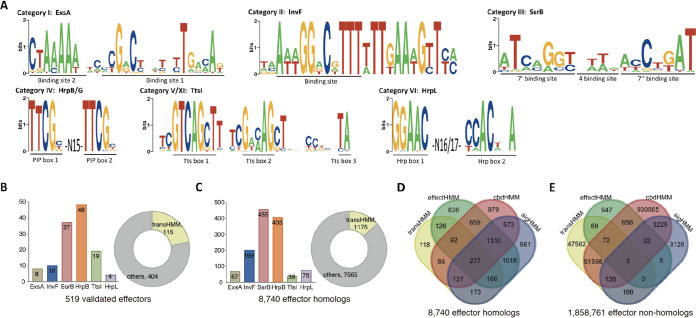
Binding motifs of T3SS regulators and their application in effector identification. (A) Binding motifs of pivotal transcription regulators of T3SS genes. The most conserved sites are indicated, with names according to previous studies (Table S1). Screening the regulatory motifs in the promoters of genes encoding validated effectors (B) and full-length effector homologs (C). The numbers of proteins screened with individual regulatory motifs are shown in the bars, and the total proteins detected with one or more regulatory motifs are shown in the circles. Interplay of proteins with DNA regulatory motifs, T3SS signal sequences, effector domains, and chaperone-binding β-motifs in full-length effector homologs (D) and in nonhomologs of full-length known effectors (E).

We previously identified 10 pivotal regulators of T3SSs from different bacterial species (5). The binding motifs for these well-studied regulators were annotated from the literature and used to train HMMs (Fig. 5A; see also Table S1). This module (transHMMs) was used to screen for corresponding regulons in species that have a T3SS(s).

10.1128/mSystems.00288-20.5 TABLE S1 Annotation for the binding motifs of pivotal T3SS transcription factors. Download Table S1, XLSX file, 0.1 MB. Copyright © 2020 Hui et al. 2020 Hui et al. This content is distributed under the terms of the Creative Commons Attribution 4.0 International license.

Only 22% (115/519) of the validated effectors had one or more of the T3SS binding motifs within their gene promoter regions (Fig. 5B; Data Set S1, sheet 7). A smaller percentage of the full-length effector homologs showed these DNA motifs (1175/8740 [13%]) (Fig. 5C; Data Set S1, sheet 7). However, nearly all of these full-length homologs with DNA regulation motifs (1057/1175 [90%]) were simultaneously detected with at least one of the other protein-specific features (Fig. 5D; Data Set S1, sheet 7). Two hundred seventy-seven contained all four conserved sequence features: DNA regulation, signal domains, CBD motifs, and effector domains (Fig. 5D; Data Set S1, sheet 7).

The transHMMs module was also applied to the 1,858,761 effector nonhomologs, screening for regulation motifs in the upstream regions (with 2-kb length) of protein-encoding genes of T3SS-bearing strains. However, very few of the proteins (448) were meanwhile detected with signal domains and/or effecting domains (Fig. 5E; Data Set S1, sheet 7). Only 3 proteins were detected with the DNA motifs, signal domains, CBD motifs, and effecting domains simultaneously (Fig. 5E; Data Set S1, sheet 7).

### New machine learning-based T3SS effector prediction models.

Within T3SE protein sequences, there can be atypical features buried in the signal sequence as well as adjacent regions that are not easily detected by homology screening. Therefore, we retrained two previously developed models with updated effector data sets, analyzing the position-specific amino acid composition (BPBAac [16]) and adjacent-residue constraints (T3_MM [18]) in T3S signal sequences to identify proteins with these atypical features (Table 1). We also trained BPBent, a modified version of BPBAac that calculates individual relative entropy of position-specific amino acid composition between effectors and noneffectors. Thirty sequential AAC features were identified within signal sequence regions with striking difference between verified effectors and noneffectors, and they were used for development of SeqAac, a decision tree model (see Table S2). Finally, two deep learning models, together named T3SEdl, one with fully connected neural networks (DNN) and the other with convolutional neural networks combined with long short-term memory cells (CNN-LSTM), were trained to compare the hierarchical amino acid composition features within T3S signal sequences. Among the models, T3_MM performed best. For the 10-fold cross-validated receiver operating characteristic (ROC) curves, the average area under the curve (AUC) reached 0.91 (Table 1). Another T3SE prediction model, ANN, was also retrained with the data set curated in this research, and the performance was not comparable to that of T3_MM or T3SErnn (see Fig. S2). The models considered different profiles from various aspects of T3SE signal sequences, and an ensemble model would be desired that take the pieces of atypical features together to make more precise prediction. T3SEppML, for which a voting-based method was adopted to integrate the individual models by counting the models with positive predictions, generated better performance with an average AUC, Matthews correlation coefficient (MCC), and accuracy (ACC) of 0.96, 0.74, and 0.90, respectively (Table 1).

**TABLE 1:** Newly, retrained, and integrated models predicting T3SEs

Model	Feature	Algorithm^*a*^	Tenfold cross-validation value (mean ± SD)^*b*^		
			AUC	MCC	ACC
BPBAac	Position-specific AAC	SVM	0.824 ± 0.068	0.524 ± 0.114	0.760 ± 0.056
BPBent	Relative entropy of position-specific AAC	SVM	0.818 ± 0.069	0.492 ± 0.147	0.744 ± 0.073
T3_MM	Conditional probability on adjacent residue	MM	0.907 ± 0.039	0.708 ± 0.102	0.850 ± 0.053
SeqAac	Sequential AAC	DT	0.758 ± 0.032^*c*^	0.523 ± 0.068	0.758 ± 0.032
T3SEdnn	Hierarchical AAC	DNN	0.859 ± 0.001	0.539 ± 0.005	0.870 ± 0.001
T3SErnn	Hierarchical AAC	CNN-LSTM	0.891 ± 0.001	0.617 ± 0.006	0.866 ± 0.001
T3SEppML	All of the above	Voting	0.960 ± 0.010	0.740 ± 0.065	0.904 ± 0.013
T3SEpp	Unified	LM	0.976 ± 0.005	0.876 ± 0.012	0.941 ± 0.011

a SVM, support vector machine; MM, Markov model; DT, decision tree; LM, linear model.

b AUC, area under the (receiver operating characteristic) curve; MCC, Matthews correlation coefficient; ACC, accuracy.

c The AUC of SeqAac was approximated by the accuracy because the prediction results were not continuous.

10.1128/mSystems.00288-20.2 FIG S2 Performance comparison among T3_MM, T3SErnn, T3SEdnn, ANN, and SVM models. Tenfold cross-validation results were based on and ANN and SVM models were retrained based on Löwer and Schneider (15). Download FIG S2, PDF file, 0.1 MB. Copyright © 2020 Hui et al. 2020 Hui et al. This content is distributed under the terms of the Creative Commons Attribution 4.0 International license.

10.1128/mSystems.00288-20.6 TABLE S2 Sequential features used for SeqAac models. Download Table S2, XLSX file, 0.1 MB. Copyright © 2020 Hui et al. 2020 Hui et al. This content is distributed under the terms of the Creative Commons Attribution 4.0 International license.

**FIG 6:**
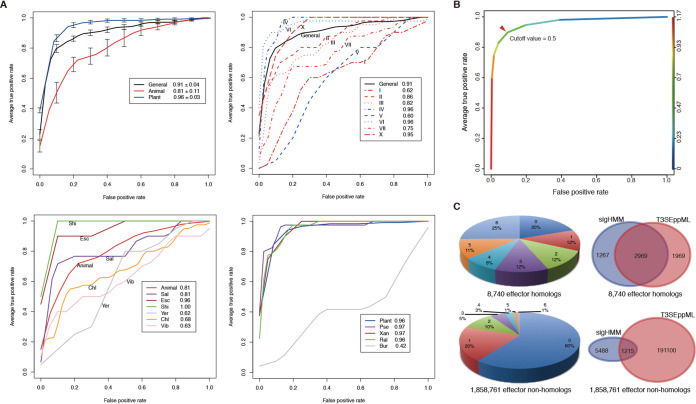
Performance of cluster-specific models and the voting-based ensemble model on prediction of T3S signal sequences. (A) Tenfold cross-validated receiver operating characteristic (ROC) curves of models categorized by bacterial host (top left), T3SS cluster (top right), animal-microbial genus (bottom left), and plant-microbial genus information (bottom right). (B) Tenfold average ROC curve of the integrated model T3SEppML and the optimization of cutoff values. (C) Prediction of T3S signal sequences from full-length effector homologs (top) and nonhomologs (bottom). The pie charts represent the proportion of proteins identified by machine learning models. The category number (0 to 6) indicates the number of models predicting the proteins. The Venn diagrams show the overlap in proteins detected with sigHMM and T3SEppML signal sequence models.

To further test whether T3SEs can be recognized more accurately with more specific prior information, we retrained T3_MM with an integration of animal or plant pathogen/symbiont, T3SS group, and bacterial genus information according to Hu et al. (5). As shown in Fig. 6A, the plant microbe-specific model outperformed the animal model (AUC, 0.96 versus 0.81) (Fig. 6A, top left). We also retrained a T3SS category-specific model for each T3SS group with more than 10 nonredundant validated effectors (I to VII and X; for T3SS categories and their representative T3SSs, refer to http://www.szu-bioinf.org/T3Enc/browse_t3ss_type.html) (5). Models IV and VI, which are two large groups exclusively composed of T3SSs from plant-related microbes, performed the best, reaching 0.96 for AUCs (Fig. 6A, top right). There was also a small group (V) that was composed of the T3SSs of plant symbionts (*Rhizobium* and others), for which the model performance was relatively poor (AUC, 0.60). For groups composed of T3SSs from animal microbes, however, model performance varied a lot. One small group (X) composed of the chromosome II T3SSs of *Vibrio* showed the best model performance (AUC, 0.95), three large groups showed moderate performance (AUCs of 0.86 for II, 0.82 for III, and 0.75 for VII), whereas a model based on the other large group performed poorly (AUC, 0.62 for I) (Fig. 6A, top right). We also developed genus-specific models for each genus with more than 10 nonredundant validated effectors (including *Shigella*, *Escherichia*, *Salmonella*, *Chlamydia*, *Yersinia*, *Vibrio*, *Pseudomonas*, *Xanthomonas*, *Ralstonia*, and *Burkholderia*). Performance of the models for animal genera showed large variance (Fig. 6A, bottom left) compared to that of the models of plant genera, which were much more uniform (Fig. 6A, bottom right). The *Shigella*- and *Escherichia*-specific models performed best among the animal genus-specific models (Fig. 6A, bottom left, Shi and Esc), and the effectors from these two genera are known to be secreted through a single group of T3SSs. By comparison, in the two animal models with the poorest performance (Fig. 6A, bottom left, Vib and Yer), the effectors are known to be secreted through two different groups of T3SSs (38, 39). The *Burkholderia*-specific model was the exception among the plant genus-specific models, with deteriorated performance (Fig. 6A, bottom right, Bur), but it was trained with only a few validated effector sequences.

Despite the advantages of T3SE prediction with bacteria/T3SS-specific models for many groups, the paucity of validated effectors reduces their effectiveness. Therefore, we used the general (nonspecific) models to predict proteins with atypical T3S signal features from the T3SS-bearing bacterial proteomes. The integrated model T3SEppML was used to make predictions with an optimized cutoff value of 0.5, by which three of the six models copredicted a protein to contain T3S signals (Fig. 6B). Eighty percent (7,017/8,740) of the full-length effector homologs were predicted by at least one machine learning model to contain a T3S signal sequence, and 56% (4,938/8,740) were predicted by T3SEppML to be T3SE candidates above the target cutoff (Fig. 6C, top left). Only 10% of the effector nonhomologs were detected with T3S signals, however, the absolute number reached 192,215, since the total number of nonhomologs was huge (Fig. 6C, bottom left). Both sigHMM and T3SEppML were used to analyze the atypical features buried in T3S signal regions; these two models identified 2,969 (∼two-thirds) of the same proteins that were identified as effector homologs by flBlast (Fig. 6C, top right), and yet they showed large complementarity between each other. For the effector nonhomologs, the overlap between sigHMM and T3SEppML prediction hits was much smaller, representing only 0.06% of the total proteins screened (Fig. 6C, bottom).

### Unified pipeline to predict T3SS effectors weighting homology, machine learning results, and other biological features.

**FIG 7:**
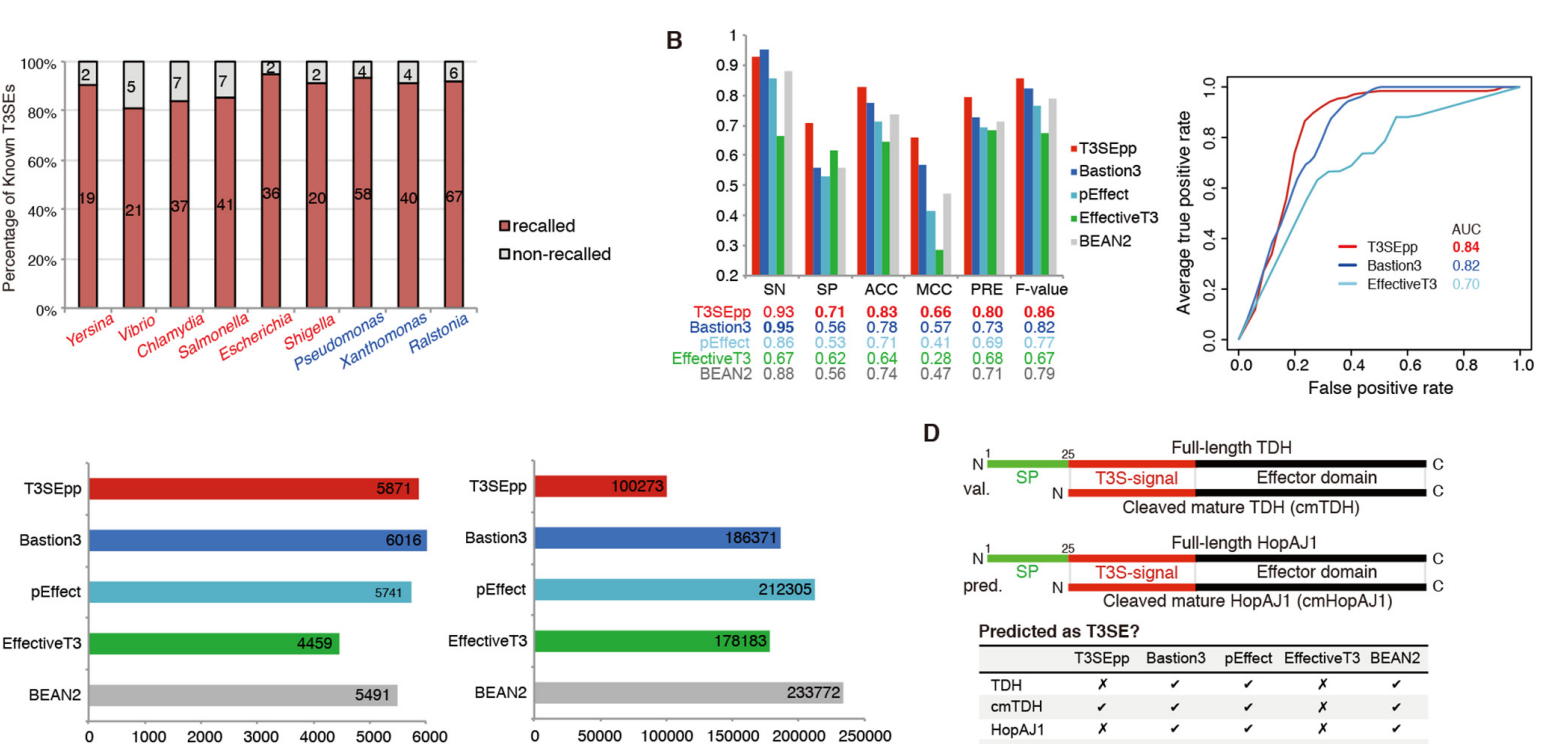
Prediction performance of T3SEpp. (A) Cross-genus prediction effect of T3SEpp with a leave-one-genus-out (LOGO) strategy. (B) Classification performance of T3SEpp and other machine learning models for independent testing data set. The ROC curves are shown on the right. SN, sensitivity; SP, specificity; ACC, accuracy; PRE, precision. (C) T3SE candidates predicted by different tools from full-length homologs of verified T3SEs (left) and nonhomologs (right). (D) Examples correctly predicted by T3SEpp rather than other tools. The protein structures of full-length and cleaved mature TDH (val, validated) and HopAJ1 (pred, predicted) are shown. The table at the bottom shows the prediction of TDH and HopAJ1 by different tools.

We developed an integrated pipeline (T3SEpp) to consider the multi-aspect and multifragmental signatures of T3SEs simultaneously, with the goal to improve the prediction accuracy and reduce the false-positive rate. T3SEpp is a linear model that weighs the contribution of each T3SE prediction module described above (i.e., both homology-based and machine learning models). The model architecture and optimized weights are described in Materials and Methods (and at http://www.szu-bioinf.org/T3SEpp/modules.html). The prediction performance was improved, with 10-fold cross-validated AUC reaching 0.976 (Table 1). Leave-one-genus-out (LOGO) assessment demonstrated the superior performance of T3SEpp in cross-species prediction, with a recall rate of greater than 80% for the known T3SEs in various genera (Fig. 7A). Similar to previous results (Fig. 6A), the recall rates of animal pathogens (Fig. 7A, red genera) varied more than those of plant pathogens (Fig. 7A, blue genera).

We also compared the performance of T3SEpp with that of other state-of-the-art software tools, including EffectiveT3 (13), BEAN2 (21), pEffect (32), and Bastion3 (23). DeepT3 (24) was not included because of a failure in running it on a local system. From the literature, we compiled an independent testing data set, containing 42 new T3SEs and 34 non-T3SEs that were experimentally validated to be translocated or nontranslocated through T3SS conduits. Bastion3 showed the highest sensitivity (95% [40/42]) but was only slightly higher than T3SEpp (93% [39/42]) (Fig. 7B). However, T3SEpp showed increased specificity (71% [24/34]), precision (80% [39/49]), and accuracy (83% [63/76]) compared to those of Bastion3 and the other tools (Fig. 7B). Another independent data set from Xue et al. (24) was tested, which also yielded higher specificity, accuracy, MCC, and F-value for T3SEpp than for Bastion3 and pEffect (see Fig. S3). To further demonstrate the main advantage of T3SEpp (i.e., high specificity and sensitivity), we screened the 8,740 full-length T3SE homologs and 1,858,761 T3SE nonhomologs. Although Bastion3 recalled slightly more homologous T3SEs than T3SEpp (6,016 [69%] versus 5,871 [67%]), it predicted almost double the T3SEs from the nonhomologous protein set (186,371 versus 100,273) (Fig. 7C).

10.1128/mSystems.00288-20.3 FIG S3 Prediction performance of T3SEpp and other tools using testing dataset by Xue et al (24). SN, sensitivity; SP, specificity; ACC, accuracy; PRE, precision. Download FIG S3, PDF file, 0.8 MB. Copyright © 2020 Hui et al. 2020 Hui et al. This content is distributed under the terms of the Creative Commons Attribution 4.0 International license.

To illustrate the differences between T3SEpp and other prediction tools, we tested the performance on two proteins, thermostable direct hemolysin (TDH) from Vibrio parahaemolyticus and HopAJ1 from Pseudomonas syringae (Fig. 7D). TDH is secreted through a type II secretion system (T2SS) and a T3SS in Vibrio parahaemolyticus (40). The full-length TDH protein contains an N-terminal signal peptide that guides the Sec pathway secretion (i.e., T2SS), while the form of TDH with the Sec signal peptide cleaved (i.e., cmTDH) exposes an N-terminal T3S signal sequence and is then translocated through the T3SS conduit. Therefore, full-length TDH is not a true T3SE, while cmTDH is a T3SE (both being validated by translocation assays) (40). With T3SEpp, full-length TDH was correctly identified as non-T3SE and cmTDH was identified as a T3SE, whereas none of the other tools were able to make this distinction (Fig. 7D).

HopAJ1 is a T3SS coregulated gene in P. syringae pv. *tomato* DC3000 with a predicted lytic transglycosylase domain (41). Although T3SS translocation assays were negative for this protein (41), all of the software prediction tools identified HopAJ1 as a T3SE, except T3SEpp (Fig. 7D). We also identified a putative ∼25-amino-acid signal peptide at the N terminus of HopAJ1 (Fig. 7D). Interestingly, when the truncated version of HopAJ1 (i.e., cmHopAJ1) was screened, T3SEpp software identified it as a T3SE (Fig. 7D). The cmHopAJ1 protein possesses a T3 signal sequence successfully predicted by 5 of the 6 machine learning models compared to only 1 of 6 models of positive prediction for the full-length HopAJ1 (http://www.szu-bioinf.org/T3SEpp/20200329.php). Based on these findings, we hypothesize that HopAJ1 could be secreted by P. syringae in a way similar to that of TDH in V. parahaemolyticus.

## DISCUSSION

We have developed a unified T3SE prediction pipeline, T3SEpp, consisting of multiple modules to incorporate a wide variety of T3S sequence-based features. The result is a highly sensitive and specific prediction software that is improved compared to other state-of-the-art software. We are introducing a webserver to implement the T3SEpp pipeline efficiently and that also provides the analysis results of the individual modules. The standalone version of T3SEpp and individual modules can also be downloaded and used on a local system independently (http://www.szu-bioinf.org/T3SEpp/download.html).

When the individual prediction modules that comprise T3SEpp are used in isolation, they can successfully identify T3SEs, but they also predict many false positives. For example, we know that T3SE signal sequences are important, and these are usually mined as common features for T3SE prediction. Since the introduction of machine learning techniques for prediction of bacterial type III secreted effectors (13, 14), more than a dozen software tools have been developed, and these most frequently rely on the N-terminal signal region. However, the signal regions are only necessary but not sufficient for T3SS translocation. In this study, we unexpectedly found high sequence similarity among known effectors, especially in the N-terminal signal regions. It is our hypothesis that this undetected homology in the training data sets could cause bias and lead to severe overfitting in the established machine learning algorithms. In our experience, there was often a large discrepancy between the reported accuracy and the practical accuracy of these methods. On the other hand, there is potential for the N-terminal homology we detected among T3SS effectors to be useful in the identification of new effectors (5, 27).

Clustering the N-terminal signal sequences from 519 verified effectors based on homology generated 379 signal sequence families. In general, there was not great consistency between signal sequence families and full-length effector families. This was not surprising because terminal reassortment happens frequently among T3SS effectors (26, 27). The low occurrence of verified effectors for both conserved domains and signal families further indicated the possibility of frequent reassortment events (see Data Set S1, sheet 5, in the supplemental material). The variety of effector proteins could be further broadened by the combinations with distinct T3S signal patterns and effector domains, among which 424 unique clusters were detected (Data Set S1, sheet 5). The conservation of β-motifs in chaperone-binding domains also facilitated the identification of new effectors with increased specificity. However, only 46% of the verified effectors appeared to have this β-motif (Fig. 3B), suggesting that the remaining effectors could have a different motif, a degenerate one, or even no such motifs. On the other hand, many other proteins that we screened contained the β-motif but did not show homology to full-length known effectors or T3SS signal sequence regions. This indicated that screening for a β-motif in isolation could lead to a large number of false positives. We concluded that β-motif screening must be combined with that for other sequence features to increase model precision but has little contribution to the overall predictive power.

A large number of proteins (1,784) were predicted based on homology to known T3SS effector domains (Fig. 4B and D) but remained undetected by full-length homology screening. This demonstrated the advantage of incorporating effector domain homology searching in T3SE prediction. Very few of these proteins contained both putative T3SS signals and effector domains (Fig. 4C and D), which supports the independent evolution hypothesis of T3SS signal families and functional domain families (26, 27) and highlights the value of fragmental homolog detection algorithms. We hypothesize that some proteins with putative effector signal sequences but without known effector domains could represent new effector domain families, especially for proteins that also contained CBD motifs.

The timed release of T3SS effectors is a product of fine and coordinated regulation of gene expression. We previously identified that T3SS gene regulation displays high-level diversity and low-level conservation (5). A typical T3SS often has a pivotal regulator that directly regulates the expression of the effector genes. Many of the pivotal regulators from different T3SSs belong to the same transcription factor families, which share similar promoter binding motifs (5, 42). Identification of DNA regulatory motifs can assist in finding new effector genes (30, 31), but when this module (i.e., transHMM) was used alone, it showed only limited sensitivity. This low sensitivity can likely be attributed to the lack of knowledge about other T3SS regulators as well as the evolution or degeneracy of promoter binding motifs. However, positive detection of these motifs would improve the precision of effector recognition.

Despite a large number of false-positive predictions, machine learning algorithms still have advantages for the identification of atypical features and prediction of novel effector candidates. In this study, we newly trained or retrained six machine learning models, each focusing on different feature profiles of T3S signal sequences. T3_MM, which is a Markov chain model based on amino acid composition probability conditional on the amino acid composition at the preceding position within the N-terminal region (13), performed the best in our hands; however, other models should also have their own merits for identifying specific atypical T3S signal features. Therefore, we developed an ensemble model, T3SEppML, to make an integrated consideration of the results from the six different models. T3SEppML outperformed each of the individual models (Table 1). When we compared T3SEppML and sigHMM, we concluded that although T3SEppML was capable of identifying novel T3S signal sequences, the numbers of false positives remained a big problem for whole-genome predictions. In our estimation, instead of making the programs more stringent, it was better to combine them with modules that identify features in regions outside the T3S signal sequences.

We also tested the potential influence of prior clustering information on model prediction performance, e.g., bacterial host, T3SS cluster, and bacterial evolution. We found that performance varied a lot with T3SS categories derived from animal-associated microbes, whereas the plant-associated microbes were more consistent, with only a few exceptions. This could be due to several animal-associated microbes containing multiple T3SSs (5). Although it is possible for genus-specific models to be improved in the future, it is not possible to retrain the models due to a limitation in the numbers of validated effectors.

Although it remains a challenge to predict T3SEs accurately, especially when using whole genomes, we hypothesize that combining multi-aspect features is the best way to reduce the rate of false positives. Accordingly, our T3SEpp software had the lowest false-positive prediction rate compared to those of other state-of-the-art software tools (i.e., Bastion3, EffectiveT3, BEAN2, and pEffect) when tested against an independent data set containing experimentally validated T3SEs and non-T3SEs. For the full-length effector homologs, T3SEpp showed similar positive prediction rates with those of other tools; however, T3SEpp predicted nearly 1-fold fewer T3SEs from the nonhomologs. T3SEpp can also disclose many important biological features for the T3SEs or candidate proteins, including the T3S signal family, effector domain family, presence of a chaperone binding motif, and putative regulation by key T3SS transcription factors. For example, T3SEpp was the only tested tool that correctly classified full-length TDH as a non-T3SE and mature TDH as a T3SE (Fig. 7D). The software also correctly predicted full-length HopAJ1 to be a non-T3SE and indicated that loss of a putative Sec signal peptide might allow the truncated HopAJ1 protein to become a functional T3SE (Fig. 7D). To this end, it would be interesting to examine if HopAJ1 can be secreted by P. syringae in a mechanism similar to that for TDH in V. parahaemolyticus.

Despite the advances with the T3SEpp model described here, there are still many features that can be improved. Better precision involves the understanding of the length, position, and key residue composition of T3S signal sequences and/or chaperone-binding domains. Similarly, it is a problem to select the proper negative training data sets with respect to protein size and composition. New features such as the phylogenetic conservation of proteins or the genomic adjacency of effectors and chaperones could be further examined and integrated to improve the prediction precision. Other aspects related to the specificity of T3SEs (e.g., bacterial host, T3SS cluster, and bacterial genus or species) are dependent on the validation of more T3SEs. As this information becomes available, the prediction accuracy could be further improved.

### Conclusions.

To our knowledge, we have compiled the most comprehensive biological sequence feature analysis for T3SEs in this research. The T3SEpp pipeline integrating the variety of features and assembling different models showed high accuracy, which should facilitate more accurate identification of T3SEs in new and existing bacterial whole-genome sequences.

## MATERIALS AND METHODS

### Data sets.

Five hundred nineteen prefiltered, manually annotated T3SS effectors were downloaded from T3Enc (http://www.szu-bioinf.org/T3Enc). Prefiltering was performed according to Hu et al. (5), by which effector families were identified for each bacterial genus, with only one validated effector retained in a genus for each effector family. JAligner (http://jaligner.sourceforge.net/) was applied to implement the Smith-Waterman algorithm to find the similarity between any pair of full-length or designated lengths of effector proteins. By default and not specified, the homology between two proteins was defined as ≥30% similarity for ≥70% average coverage of the full length. The identity or similarity percentage between any pair of sequences was recorded and compared with the cutoff defined in context to determine the homology level. To retain the most clusters after homology filtering, a recursive hub-filtering algorithm was developed, by which the effector with the largest number of homolog edges was removed from the homolog network recursively until no edge remained. For the full-length verified effectors or fragments, the overall interprotein homology was measured by the number of homologous clusters, which was reversely related with the overall homology. For example, with the same homology-clustering criteria, clustering into 519 homologous groups would suggest an apparently lower homology among proteins than those clustered into only one homologous group. EBT, a statistical method similar with but showing higher precision than chi-square or Fisher’s exact test, was applied to compare the interprotein general homology (43). For the bacterial strains of verified effectors, the genome-derived proteome was filtered for known effectors. In total, 2,000 proteins with more than 100 amino acids (aa) in length were selected randomly from the remaining proteins of the strains to form the parental negative data set. BLAST was used to align the verified effector sequences against the parental negative protein sequences, and the possible effector homologs were removed if the similarity was higher than 30% for larger than 70% average aligned protein length (https://blast.ncbi.nlm.nih.gov/). The remaining full-length or designated-length proteins were compared with JAligner and those with intra- or inter-data set homology were removed. The proteins or peptide fragments surviving the homology filtering steps constituted the final negative data sets.

The 8,740 full-length homologous effectors were downloaded from T3Enc (5). The genomes and their encoding proteome sequences of bacterial strains with one or more T3SSs annotated in T3Enc were downloaded from NCBI Genome database, while the effector homologs were removed, and the remaining proteins comprised the effector nonhomolog data set.

### Homology-based T3SS effector prediction models.

Full-length homology searching and family clustering was according to Hu et al. (5), and a gene ontology (GO) program, flBlast, was developed to implement the procedure automatically. Briefly, the 519 validated full-length T3SEs were clustered into families based on similarity measured by BLASTp alignment results (https://blast.ncbi.nlm.nih.gov). Two proteins showing ≥30% similarity for ≥70% of the full length of either protein were considered to have homology (5). A network approach was used to cluster the homologous proteins into families, i.e., a protein was clustered into a family only if it showed homology with at least one member of the family, with no homology allowed between any members of any two families. All 519 proteins were collected and used as a database for sequence alignment. GO scripts were prepared to implement the BLASTp program to make alignment of candidate proteins against the T3SE database and report the most significantly homologous hit and corresponding full-length T3SE families. The homology searching criteria were same with those for effector clustering, i.e., ≥30% similarity for ≥70% of the full length of either candidate protein or any one known T3SE.

For homology searching of known T3SS effector signal sequences, the N-terminal 50-aa peptides of the verified effectors were grouped to signal clusters based on the homologous network at a minimum cutoff of 30% identity for 70% aligned protein length. The multiple peptides in each single cluster were further aligned with ClustalW to define signal families more precisely (https://www.ebi.ac.uk/). The cluster could be separated into different families if the homologous blocks showed no overlap. Each singleton cluster, i.e., with only one member that did not show above-cutoff similarity with other effector peptides, represented an independent signal family. For each multimember family, all the peptide sequences were used for building the family profile. For each singleton family, the orthologs from other strains of the same genus were retrieved, and the N-terminal 50-aa peptides were aligned against the counterpart of the reference singleton sequence; only the ones with at least 70% identity for the whole 50-aa length were retained and used for the family profile building. The HMM profile of each signal sequence family was built with the hmmbuild module of HMMER 3.1, followed by alignment and homology searching with hmmsearch (44). A GO program (sigHMM) was also developed to implement the T3S signal searching, with the cutoffs (hmmsearch E values) optimized by allowing all the signal-bearing validated effectors being detected.

Lilic et al. first reported a common structural β-motif located within CBD domains, where the sequences were downloaded and aligned for HMM profile building (37). HMMER 3.1 was also used to build the HMM profile for the CBD motif sequences (44). Intraprotein location analysis indicated that the β-motifs mainly started from the N-terminal 21st to the 90th amino acid of known effectors; therefore, only the N-terminal 100-aa peptide fragment of a candidate protein was extracted for β-motif screening, using a GO program developed specifically (cbdHMM) to implement the hmmsearch (with default parameter) and pattern search automatically. Secondary structure modeling and alignment for the β-motifs were performed with PROMALS3D (45).

N-Terminal 50-aa signals were also removed from each known effector protein sequence, and the remaining peptide fragment was used for effector domain clustering if it was longer than 30-aa. Pairwise alignment was repeatedly performed with BLAST among the domain sequences of known effectors, and a homologous pair was defined with optimization as average protein coverage of aligned length × identity of ≥10. A recursive algorithm was adopted to cluster the families (5). HMM profiles of the effector domain families were also built with HMMER 3.1, and the screening of family homologs was performed using a similar procedure as described above with a modified E value cutoff of 0.01. A GO program, effectHMM, was developed for implementing effector domain searching automatically.

Pivotal transcription regulators of T3SSs were annotated previously (5). The binding motifs within promoters of the regulated genes were annotated from the literature. HMM profiles or sequence patterns were built. To screen these motifs or patterns, the genome sequences of representative bacterial strains with T3SSs were downloaded from NCBI Genome database, the 5′ upstream 2,000-nucleotide sequence preceding the effector open reading frame (ORF) was retrieved, and the HMM profiles were screened one by one. Motif patterns were also screened with a pattern recognition method implemented in an in-house GO programming script (transHMM). For promoter motif searching, the true promoter sequence of a candidate gene is the best choice. The 5′ upstream 2,000-nucleotide sequence of the operon is the secondary choice if the exact promoter is unknown. However, for many cases, it could also be unclear for operon organization, and the 5′ upstream 2,000-nt nucleotides preceding the target gene are the only choice. In this study, for convenience of genome-wide analysis, the third choice was applied.

### Data set preparation for training and performance evaluation of machine learning models.

For general models, the N-terminal 100 amino acids were retrieved from the verified effectors and noneffector proteins with length larger than 100 amino acids (with the starting methionine removed), followed by homology filtering with JAlign using the strategy described above and the homology criterion of 30% identity for whole length. The procedure led to 309 nonredundant effector signal peptides, which served as the positive data set. From the nonredundant noneffector peptides, 310 were selected randomly to serve as negative data set, making an ∼1:1 ratio of sequence size between positive and negative data sets. Both the positive and negative data sets were randomly split into 10 parts with identical numbers of sequences. For each 10-fold cross-validation, nine parts of the positive and negative data sets were merged as positive and negative training data sets, respectively, while the remaining one part of either the positive or negative data set was used as the corresponding testing data set.

For bacterial host-, T3SS-, or genus-specific models, the homology-filtered signal sequences were retrieved from the corresponding group, and an identical number of homology-filtered negative sequences were sampled from strains of the corresponding group. Bacterial hosts, T3SS category, and genus information were referred to T3Enc (5). Tenfold cross-validation training and testing data sets were prepared as described above.

### Modified BPBAac, BPBent, T3_MM, and SeqAac models classifying proteins containing or not containing type 3 secreted signals.

Feature representation, parameter optimization, and model training for BPBAac and T3_MM were according to the procedure described previously (16, 18). Briefly, BPBAac represented the N-terminal 100-aa sequences in vectors with biprofile position-specific amino acid composition features extracted from both positive and negative training data sets, which were further trained in support vector machine (SVM) models. Four SVM kernel functions, i.e., linear, polynomial, sigmoid, and radial base function (RBF), and corresponding parameters (gamma and cost), were all optimized with a 10-fold cross-validation grid search strategy. An R package, e1071, was used for implementing SVM model training and kernel/parameter optimization (https://cran.r-project.org). T3_MM calculated the probability values of any species of amino acids conditional on the amino acid composition in its adjacently preceding position for both positive and negative N-terminal 100-aa training sequences. For each sequence, two Markov chains were built with each amino acid represented with the conditional probability values extracted from the positive or negative training data set, followed by calculation of the logarithm likelihood ratio (positive/negative), which was used for eventual classification. The cutoff was adjusted to find an optimal solution for both specificity and sensitivity.

For BPBent, instead of position-specific AACs, the individual relative entropy was calculated for position-specific AACs between T3S signal sequences and total bacterial proteins, between non-T3S sequences and total bacterial proteins, and between T3S signal sequences and non-T3S sequences. The triprofile relative entropy values were represented as a vector for each sequence, and the feature matrixes were trained with SVM models as performed for BPBAac (16). The kernel selection and parameter optimization were also similar to the procedure for BPBAac as described above.

For SeqAac, in total, 431 sequential features were observed and compared between T3S and non-T3S sequences, including composition of individual amino acids (20), biresidues (20 × 20 = 400), and amino acids of 11 specific properties (aliphatic, aromatic, hydrophobic, alcohol, polar, tiny, small, bulky, positively charged, negatively charged, and charged) (see Table S3 in the supplemental material). Bonferroni-corrected Student’s *t* tests were performed between the sequential composition of features between T3S and non-T3S sequences, and the top 30 features were selected for model training (Table S2). For each sequence, the composition of each feature was calculated and put into a vector. The matrix composed by the 30-feature vectors for each training data set was used for a decision tree building and pruning with the R rpart package (https://cran.r-project.org).

10.1128/mSystems.00288-20.7 TABLE S3 All sequential features observed in this study. Download Table S3, XLSX file, 0.1 MB. Copyright © 2020 Hui et al. 2020 Hui et al. This content is distributed under the terms of the Creative Commons Attribution 4.0 International license.

### Deep learning models predicting type 3 secreted signals.

The T3SErnn model used in this study is composed of convolution layers, recurrent layers, and fully connected dense layers. The architecture used in our model is summarized as follows: the input is a 100 × 20 matrix, where 100 is the length of sequence and 20 is the size of amino acid vocabulary. The convolutional neural network extracts motif information using 30 filters with different sizes (5 for each of the sizes 1, 3, 5, 7, 9, and 11). By concatenating the above-described features, we obtained a 120 × 5 feature map. Then, we applied another convolution layer with 50 filters of size 3 × 120 to this feature map and obtained a 120 × 50 feature map as input to the recurrent layer. The recurrent neural network scans the above-described feature map using 256 LSTM units in two directions and returns the last output in the output sequence. Finally, the concatenation of the outputs of the recurrent neural network is used as input to a fully connected dense layer with 512 units and outputs the final prediction score. The parameters are optimized using adaptive moment estimation (Adam) with cross entropy as the loss function.

The T3SEdnn model is composed of three components, i.e., the input layer, the hidden layers, and the output layer. The input of the model is the stretch of a 100 × 20 one-hot matrix. Then, the model projects the input feature into new spaces layer by layer. In particular, the model includes five hidden layers with 1,024, 512, 256, 128, and 10 units. The output layer of the model is a logistic repression classifier with a sigmoid function as activation function. The parameters are optimized using root mean square propagation (RMSProp) with cross entropy as the loss function.

Both deep learning models were trained and implemented in Python3. The models are freely accessible at http://www.szu-bioinf.org/T3SEpp/download.html. The current T3SEdl models were trained on N-terminal 100-aa sequences of T3SEs and non-T3SEs and therefore only support the prediction of input protein sequences with no fewer than 100 amino acids.

### T3SEpp model and weight optimization.

For external modules PSORTb 3.1, TMHMM 2.0, and SignalP 4.1, the web-based versions were used with default settings (except for SignalP with sensitive mode) (34–36). A linear model was built to integrate the predictive results of different modules in an empirical architecture considering signal sequences (sigHMM/T3SEppML), transcriptional regulatory motifs (transHMM), effector domains (effectHMM/flBlast), chaperone-binding motifs (cbdHMM), and other subcellular location information (SignalP, PSORTb, and TMHMM). The factor value was set as 1 for a module if there was a positive prediction or 0 otherwise. The positive predictions for sigHMM, effectHMM, cbdHMM, transHMM, and flBlast specified that there was at least one homologous hit with the corresponding module and cutoff described before and shown in http://www.szu-bioinf.org/T3SEpp/modules.html. T3SEppML integrated the predictive results of T3_MM, BPBAac, BPBent, SeqAac, T3SErnn, and T3SEdnn with a voting strategy, and a positive prediction was defined with a general prediction value not smaller than the optimized cutoff of 0.5 (≥3 tools predicting the candidate protein to be with a true T3S signal). For each machine learning section model (e.g., T3_MM, etc.), the parameters were optimized as described before and shown in http://www.szu-bioinf.org/T3SEpp/modules.html. For SignalP, PSORTb, and TMHMM, the positive predictions were defined as “no signal peptide” (sensitive mode), “not cytoplasmic,” and “no transmembrane domain,” respectively.

Prior weights for each module were preset empirically (sigHMM, 0.45; T3SEppML, 0.40; transHMM, 0.20; effectHMM, 0.20; T3SEppML, 0.20; cbdHMM, 0.10; SignalP, 0.05; PSORTb, 0.04; and TMHMM, 0.01). The sum of weights (*S*) for T3SEppML, transHMM, effectHMM/flBlast, cbdHMM, SignalP, PSORTb, and TMHMM was 1.00, while sigHMM weight was set as “T3SEppML weight + 0.05” and the maximum prediction was set as 1.00 for the whole model. A 10-fold cross-validation based grid search with the training data sets was performed to optimize the weight parameters. The searching space for each weight was within 0.00 to 1.00, starting from the prior settings with a step increase/decrease and the *S* constraint of 1.00. To reduce the searching space, we used a rough step increase/decrease of 0.1 to determine the boundary of optimized weights, followed by gradually decreasing the step increase/decrease to 0.05 and 0.01 to find the best-optimized parameters. For each combination of weights, the average AUC was calculated for the 10-fold cross-validated prediction results. The best-optimized parameters were those for any of which an increase or decrease did not increase the average AUC. The optimized weights are shown in http://www.szu-bioinf.org/T3SEpp/modules.html.

Besides a 10-fold cross-validation evaluation of the T3SEpp models with the training data sets curated in this study, two independent testing data sets were also prepared for performance comparison with other models. The first testing data set (T3SEpp testing data set) was curated manually from the literature and included the newly validated T3SEs and non-T3SEs that were experimentally validated not to translocate through T3SS conduits. For the detailed information on the testing data set, including the annotation, sequences, etc., refer to http://www.szu-bioinf.org/T3SEpp/download.html. Briefly, we searched “type III secretion system” or “type 3 secretion system” in PubMed and retrieved the publications posted after March of 2016. The abstracts were skimmed for each paper, and the ones with effector translocation analysis were identified for further annotation. T3SEs and non-T3SEs were all verified by translocation experiments. None of the T3SEs and non-T3SEs overlapped with the sequences in the training data set. The second testing data set was adopted from Xue et al. (24) (independent testing data set). None of the sequences from this second data set were found in the T3SEpp training or testing data sets.

### Model performance assessment and comparison.

Sensitivity, specificity, precision, F value, ROC curve, AUC, MCC, and accuracy were utilized to assess the predictive performance or compare the performance among different models. An ROC curve is a plot of sensitivity versus 1 − specificity and is generated by shifting the decision threshold. MCC considers true and false positives and negatives and is generally regarded as a balanced measure that can be used even if the classes are of very different sizes.

$$\begin{array}{l} \text{ACC}=\frac{\text{TP}+\text{TN}}{\text{TP}+\text{FP}+\text{TN}+\text{FN}}\\ \text{MCC}=\frac{\left(\text{TP}\times \text{TN}\right)-\left(\text{FN}\times \text{FP}\right)}{\sqrt{\left(\text{TP}+\text{FN}\right)\times \left(\text{TN}+\text{FP}\right)\times \left(\text{TP}+\text{FP}\right)\times \left(\text{TN}+\text{FN}\right)}}, \end{array}$$

where TP, TN, FP, and FN denote the number of true positives, true negatives, false positives, and false negatives, respectively.

A leave-one-genus-out (LOGO) strategy was also used to measure the cross-species prediction effect of the models. Briefly, the models were retrained on data sets with the sequences from a certain genus removed and used to make predictions on the left-out sequences. For T3SEpp, the different modules were retrained and optimized for the parameters. However, the weights of the integrated linear model were not optimized but used the ones of the final model.

For performance comparison of the different tools, we submitted the testing data sets to the webservers of Bastion3 (23), pEffect (32), EffectiveT3 (13), and BEAN2 (21) and predicted with the default parameters. ROCs and AUC comparison were only performed for T3SEpp, Bastion3, and EffectiveT3, since pEffect and BEAN2 do not give an overall prediction score integrating the prediction results of homology searching and machine learning modules. For Bastion3, a total score of 1 was given to replace the “-” for those experimentally validated effectors included in the training data set.

### Data availability.

GO scripts were prepared to assist implementation of the homolog screening modules. T3SEppML was developed with Perl, R, GO, and Python. The executable package and source codes are freely accessible at http://www.szu-bioinf.org/T3SEpp/download.html. A webserver was also developed with PHP to help users make on-line predictions automatically, available at http://www.szu-bioinf.org/T3SEpp.
