# Supplementary material for: T3SEpp: an Integrated Prediction Pipeline for Bacterial Type III Secreted Effectors
Source: mSystems. 2020 Aug 4;5(4):e00288-20. doi: 10.1128/mSystems.00288-20 (PMC7406222; doi:10.1128/mSystems.00288-20)
Supplement: FIG S1 [file mSystems.00288-20-sf001.pdf]

Fig S1

Yer_Ysc_YopJ	82	L	S	I	E	I	K	N	V
Yer_Ysa_YspA	31	L	A	S	S	L	T	G	V
Sal_SPI1_SopE	31	L	A	K	S	I	L	A	V
Sal_SPI2_SifA	30	L	W	E	K	I	K	D	F
Shi_Shi_OspF	39	L	Y	N	Q	I	R	Q	Y
Esc_LEE_Map	54	L	T	T	Q	L	L	Q	A
Pse_Pse_ExoU	40	L	G	V	A	L	K	S	T
Cit_Cit_NleC	52	L	Y	E	N	V	I	R	A
Vib_Chr1_VP1659	36	L	T	P	I	L	G	D	A
Vib_Chr2_VopV	26	L	Q	T	S	A	S	A	L
Edw_Edw_EseJ	24	L	S	R	G	A	S	R	V
Aer_Aer_AopO	78	L	T	E	T	L	H	A	A
Bor_Bor_BopD	89	L	M	A	V	L	Q	Q	A
Bur_T3S3_BopA	39	L	F	A	F	L	A	H	V
Pho_Pho_Cif	34	L	E	I	G	L	S	S	G
Xan_Xan_XopQ	66	L	P	A	R	L	T	P	A
Ral_Ral_RipI	60	L	R	A	K	L	R	R	V
Erw_Hrp_HrpN	82	L	G	E	G	L	S	N	A
Pan_Pan_PthG	90	L	E	A	E	L	N	N	G
Rhi_Rhi_NopC	72	L	R	S	V	A	L	Q	L
Bra_Bra_NopE2	49	L	P	A	G	A	A	V	V
**Consensus AA**		*h*	.	.	.	*h*	.	.	*h*
**Consensus SS**		e	e	e	e	e	e	e	e

Yer_Ysc_YopN	68	L	D	K	R	K	L	S	D
Pse_Pse_ExoS	24	L	G	Q	I	E	A	R	Q
Bor_Bor_BteA	86	L	N	H	W	E	A	S	Q
Mes_Mes_NopD	21	L	E	E	G	Q	A	V	Q
Pec_Pec_DspE	42	L	I	Q	Q	G	L	H	D
**Consensus AA**		*h*	.	.	.	.	*h*	.	.
**Consensus SS**		e	e	e	e	e	e	e	e

Vib_Chr2_VopL	42	L	L	G	I	P	N	L
Pse_Pse2_HopF2	37	L	T	S	I	H	Q	L
Aer_Aer_AopB	68	L	G	E	I	G	K	L
Chldo_Chldo_IncC	55	L	G	T	I	L	G	L
Rhi_Rhi_NopX	49	L	D	R	I	G	P	L
Ral_Ral_RipAX2	46	L	R	G	I	S	S	L
**Consensus AA**		*h*	.	.	*h*	.	.	*h*
**Consensus SS**		e	e	e	e	e	e	e
