# Supplementary figures and images for: T3SEpp: an Integrated Prediction Pipeline for Bacterial Type III Secreted Effectors
Source: mSystems. 2020 Aug 4;5(4):e00288-20. doi: 10.1128/mSystems.00288-20 (PMC7406222; doi:10.1128/mSystems.00288-20)

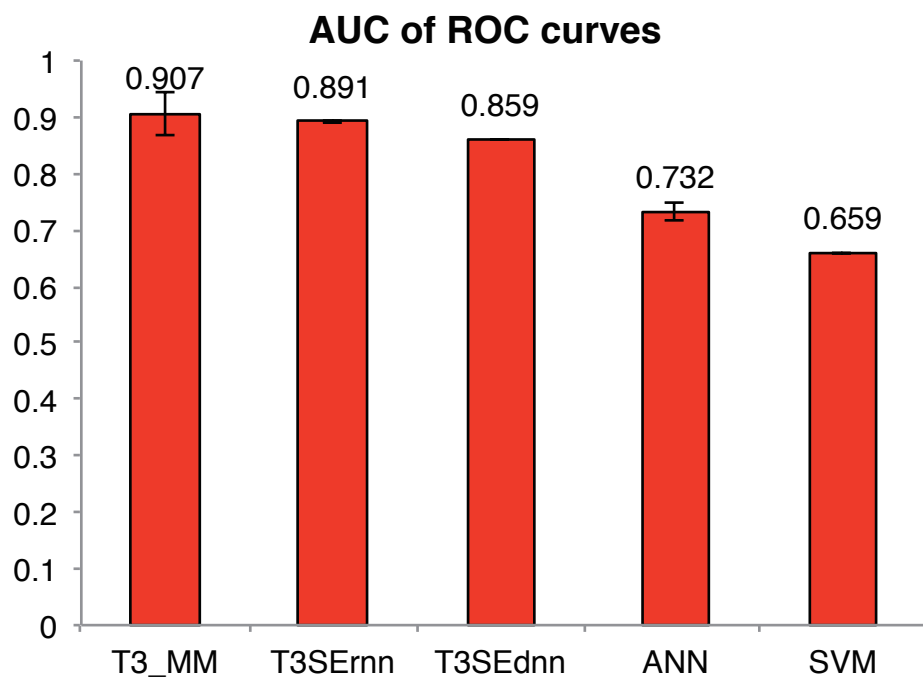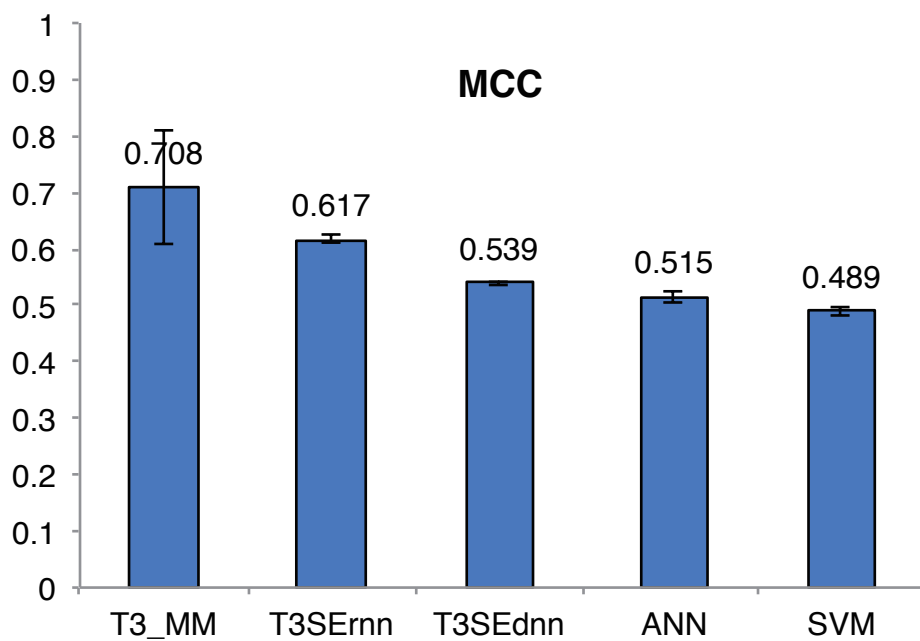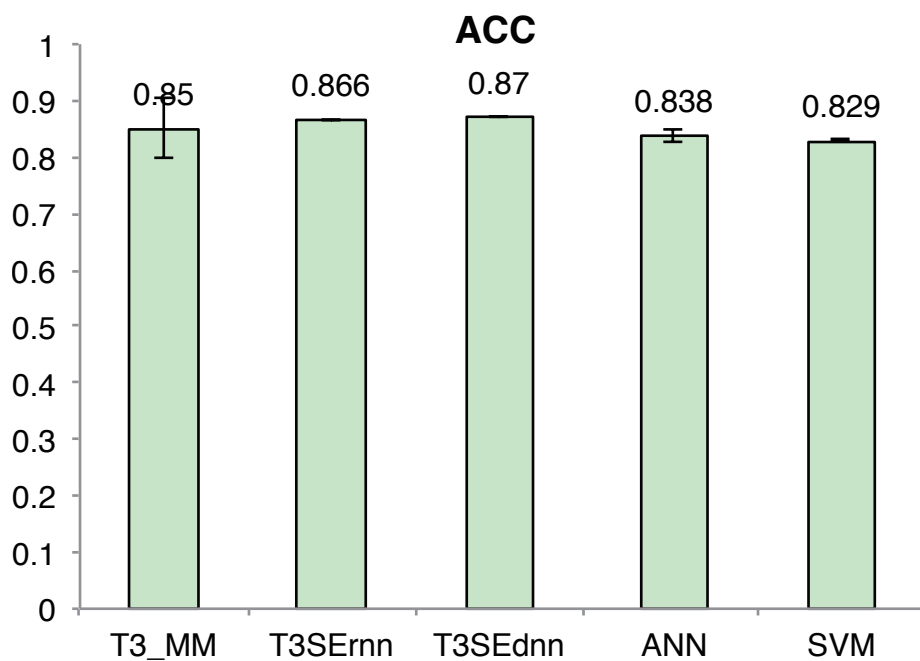

Supplement: FIG S2 [file mSystems.00288-20-sf002.pdf]

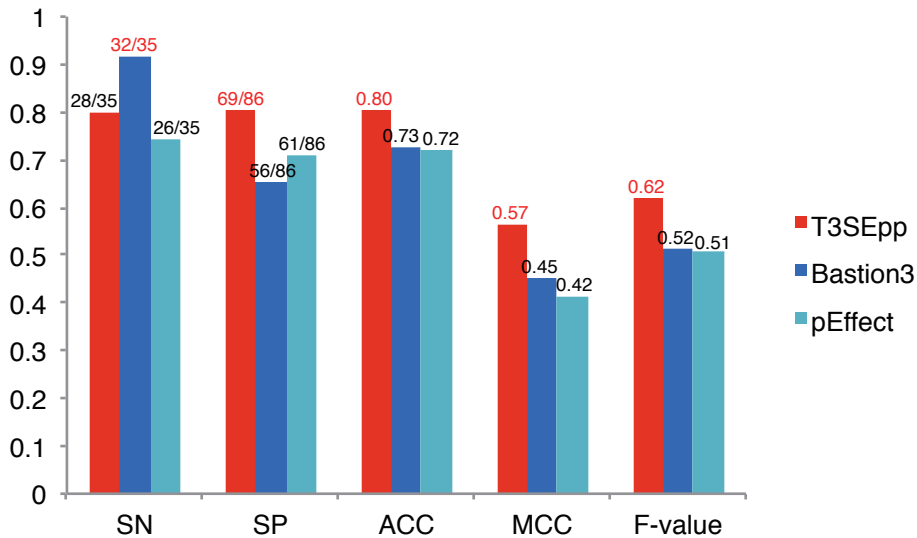

Supplement: FIG S3 [file mSystems.00288-20-sf003.pdf]
